# Which patient-reported outcomes do rheumatology patients find important to track digitally? A real-world longitudinal study in ArthritisPower

**DOI:** 10.1186/s13075-021-02430-0

**Published:** 2021-02-10

**Authors:** W. Benjamin Nowell, Kelly Gavigan, Carol L. Kannowski, Zhihong Cai, Theresa Hunter, Shilpa Venkatachalam, Julie Birt, Jennifer Workman, Jeffrey R. Curtis

**Affiliations:** 1 Global Healthy Living Foundation, Upper Nyack, NY USA; 2 Eli Lilly and Company, Indianapolis, IN USA; 3 Eli Lilly Japan, Kobe, Japan; 4 University of Alabama at Birmingham, Birmingham, AL USA

**Keywords:** Patient-reported outcomes, Real-world evidence, Rheumatoid arthritis

## Abstract

**Background:**

Patient-reported outcomes (PROs) are increasingly used to track symptoms and to assess disease activity, quality of life, and treatment effectiveness. It is therefore important to understand which PROs patients with rheumatic and musculoskeletal disease consider most important to track for disease management.

**Methods:**

Adult US patients within the ArthritisPower registry with ankylosing spondylitis, fibromyalgia syndrome, osteoarthritis, osteoporosis, psoriatic arthritis, rheumatoid arthritis, and systemic lupus erythematosus were invited to select between 3 and 10 PRO symptom measures they felt were important to digitally track for their condition via the ArthritisPower app. Over the next 3 months, participants (pts) were given the option to continue tracking their previously selected measures or to remove/add measures at 3 subsequent monthly time points (month [m] 1, m2, m3). At m3, pts prioritized up to 5 measures. Measures were rank-ordered, summed, and weighted based on pts rating to produce a summary score for each PRO measure.

**Results:**

Among pts who completed initial selection of PRO assessments at baseline (*N* = 253), 140 pts confirmed or changed PRO selections across m1–3 within the specified monthly time window (28 days ± 7). PROs ranked as most important for tracking were PROMIS Fatigue, Physical Function, Pain Intensity, Pain Interference, Duration of Morning Joint Stiffness, and Sleep Disturbance. Patient’s preferences regarding the importance of these PROs were stable over time.

**Conclusion:**

The symptoms that rheumatology patients prioritized for longitudinal tracking using a smartphone app were fatigue, physical function, pain, and morning joint stiffness.

**Supplementary Information:**

The online version contains supplementary material available at 10.1186/s13075-021-02430-0.

## Background

Developing a standardized approach to harmonize and prioritize outcome measurement in rheumatology research and clinical care has been a goal of the American College of Rheumatology (ACR), the European League Against Rheumatism (EULAR), Outcome Measures in Rheumatology Clinical Trials (OMERACT), and the International Consortium of Health Outcome Measurement (ICHOM) [1–5]. The core sets of measures developed by these groups include individual assessments and composite indices (e.g., Disease Activity Score in 28 joints [DAS28]) that incorporate use of patient-reported outcomes (PROs), as well as clinical measures reflecting clinicians’ assessments, to quantify disease activity and change over time [2]. PROs are also useful to capture patient symptoms and lived experiences that have a meaningful impact on patients’ quality of life yet would not be described as disease activity.

PRO measures are therefore important indicators of health and wellness that, ideally, convey the experience and impact of disease activity, functional limitations, symptoms, and treatment effectiveness. Identifying which outcomes are most meaningful and relevant to patients for their health decision making is a core tenet of patient-centered outcomes research, based on the premise that integration of these outcomes into research and practice will improve patients’ use of evidence in their health decisions, ultimately helping patients to better manage their disease and achieve their health goals [6–8]. As PROs are increasingly integrated with clinical measures to enhance the management and treatment of rheumatic disease [9], and in light of a growing emphasis on telemedicine and virtual healthcare resulting from the COVID-19 pandemic, little is known about the PROs that patients themselves find most important, informing which PRO measures might best augment clinical care. Due to the chronic and unpredictable nature of rheumatic diseases, it is unclear how patients’ symptom-tracking and prioritization may vary over the course of their disease and its management. Such information can help determine which symptoms best reflect the perspective of people living with rheumatic disease, informing which measures researchers and clinicians ought to heed, and those that manufacturers and medical product regulators should consider when developing PRO instruments for potential labeling claims [10–13].

This study aimed to better understand symptoms that patients with rheumatic and musculoskeletal diseases (RMD) find most important for their disease management and would be willing to track longitudinally using a smartphone app. Our intent was to supplement the existing literature around the relative importance of these symptoms from the patient’s perspective, particularly in the context of a growing need for virtual healthcare and remote patient monitoring that might be facilitated by use of a smartphone app. In this study, we examined PROs voluntarily selected by participants in the ArthritisPower registry with ankylosing spondylitis (AS), fibromyalgia syndrome (FMS), osteoarthritis (OA), osteoporosis (OP), psoriatic arthritis (PsA), rheumatoid arthritis (RA), and systemic lupus erythematosus (SLE) to elucidate which disease symptoms they consider most relevant to track digitally and which PRO measures are prioritized as most important.

## Methods

### Study design and population

This was an ancillary study to the ArthritisPower research registry (Advarra IRB protocol #00026788). ArthritisPower is a collaboration between the non-profit Global Healthy Living Foundation (GHLF) and rheumatology researchers at the University of Alabama at Birmingham (UAB). Launched in 2015, ArthritisPower comprises members with a self-reported RMD diagnosis who have provided consent to participate in research studies and provide data via the ArthritisPower app using a smartphone or web-based equivalent [14, 15]. A variety of data linkages to electronic health records, medical and pharmacy claims data, and biomarkers have been established within the ArthritisPower registry to confirm diagnoses and increase the veracity of data collection [16–18].

Members of the ArthritisPower registry who were residents of the USA or US territories that were ≥ 19 (≥ 21 for Puerto Rico residents) with a self-reported physician diagnosis of AS, FMS, PsA, OA, OP, RA, or SLE were eligible to participate in this study. Eligible members received an email invitation to participate in the “ArthritisPower Symptoms That Matter to You Study” with the goal of helping researchers and physicians better understand the symptoms that are most important to track from the patient’s perspective. After agreeing explicitly to participate in this ancillary study, participants were directed to the ArthritisPower app to select which PROs they would prefer to track and then complete the associated PRO assessments that they selected. PRO measures from physical, mental, and social health domains that were made available for selection by participants included disease-agnostic instruments developed by the National Institutes of Health (NIH) for the Patient-Reported Outcomes Measurement Information System (PROMIS) [19] or by Eli Lilly and Company [20–23] and, for participants with RA, a RA-specific measure of flare developed by OMERACT [24]. Routine Assessment of Patient Index 3 (RAPID3) [25] was considered for inclusion as an RA-specific measure familiar to most physicians, but we elected not to provide it as an option for participants due to its composite nature, which overlap with PROMIS Physical Function and pain measures, [26] and having a name that is not readily comprehensible to patients. Between the two, PROMIS Physical Function was deemed preferable as it is disease-agnostic, has a name that is easily understood by patients, and is now one of the ACR recommended measure options for functional status for RA [27]. See Appendix 1 for a complete list of PRO assessments within the ArthritisPower registry that participants were able to select from for this study.

At baseline, participants were prompted to choose which PRO instruments they wanted to track (minimum of 3; maximum of 10). This prompt was part of the in-app walk through (or “coached” input, see Appendix 2 for representative screen shots of coached input). Additional information about the nature and content of each PRO measure available for selection, as well as the estimated time for completion of each instrument, were provided to help participants make an informed decision. Specifically, participants could click a “What do these measures mean?” link on the same screen as the measure name to open a modal (pop-up) window with the supplementary information. The computerized adaptive testing (CAT) versions of PROMIS measures were offered to minimize participant burden compared to the longer short form equivalents available within PROMIS.

At three subsequent time points occurring every 28 days (i.e., month [m]1, m2, m3, each with a ± 7 day window) over this 3-month study, participants were given the option to continue tracking their previously selected PRO measures or to add, remove, and/or select different measures. At the time of study completion (m3), participants completed an exit survey to prioritize (i.e., rank) all measures selected during study participation. Specifically, participants were shown all assessments they had ever chosen during the 3 months of the study and were asked to rank 5 instruments (or the maximum number they had selected, if less than 5) in order of personal relevance (from 1 = most important to 5 = least important, with only one instrument permitted per rank order). Participants were also prompted to specify other symptoms they would have wanted to track that were not included in this study.

Participants received two subsequent monthly reminders to select/de-select their previously chosen PROs and to complete their selected PROs during the following month. Each time, they were asked to confirm whether they still wished to track their symptoms using the previously selected instruments. Participants were able to change both the number of instruments to track and which instruments those were at m1, m2, and m3. The study followed participants for a total of 3 months. Participants enrolled in the study during December and January 2018, and their participation concluded by April 7, 2019.

### Statistical analysis

Descriptive summary statistics of participant demographics were conducted for the overall cohort and for each disease subgroup (AS, FMS, OA, OP, PsA, RA, SLE). Categorical variables were analyzed by frequency counts and percentages. Continuous variables were analyzed by mean (SD), minimum, and maximum. The frequency of each PRO selected by participants overall and by condition was calculated based on observations at baseline, m1, m2, and m3, respectively. PRO ranking was based on overall frequency of selection where more frequent selection by participants meant the PRO received a higher rank. In cases where a participant completed a PRO more than once within the specified monthly window, only the first PRO was counted from that month’s window. Participants who selected more than one condition, which were not mutually exclusive, were included for analysis when comparing across monthly selections for each subgroup. For example, the RA subgroup was summarized as any participant with self-reported RA, regardless of whether reporting another rheumatic condition of interest for the study.

To calculate a weighted summary score for each PRO using completer participants’ PRO ranking at completion of the study (m3), the percentages of each rank (1 to 5 where 1 = most important, 5 = least important) were tallied based on number of observed participants selecting each PRO and its rank. Specifically, measures were rank-ordered based on the number of participants ranking it as their first, second, third, fourth, or fifth choice and weighted by multiplying the rank number by its inverse to achieve a single weighted summary score for each measure. For instance, a measure scored 1/1 if ranked as the most important, 1/2 if second most important, and so on. Values were then summed across all participants to produce a weighted summary score for each PRO measure [e.g., if an assessment was ranked first (#1) by 20 participants, second (#2) by 11, third (#3) by 15, fourth (#4) by 13, and fifth (#5) by 9, weighted summary score would be (20/1) + (11/2) + (15/3) + (13/4) + (9/5) = 35.6].

In order to compare PRO rankings across disease subgroups for valid inference, we first created mutually exclusive categories using a multipurpose hierarchy of conditions per prior ArthritisPower data analysis convention [26] where AS > PsA > SLE > RA > FMS > OA > OP. This rank order of condition categories is predicated on the idea that (a) more specific conditions are ranked higher and (b) more symptomatic conditions are ranked higher (i.e., FMS > OA). The individual participant rankings were then weighted and multiplied by 100, regardless of whether a participant had ranked the assessment (i.e., unranked PROs carried a zero value for their score) to account for a PRO’s overall popularity, and the mean of participants’ rankings of each PRO was calculated. Analysis of variance (ANOVA) for *F* statistic was conducted to compare mean PRO ranking scores overall across conditions, and then Tukey honestly significant difference (HSD) test for pairwise comparisons between condition selections on any PRO where the *F* statistic was significant at *p* < 0.05, with the variant that allows for comparison of groups with unequal sample sizes. Only PROs with mean ranking score of 10 or greater were compared. Mean scores of participants’ PRO rankings were also compared to the weighted summary score (see above paragraph) to confirm prioritization of the most important PROs for all participants. Data were analyzed as observed, with no imputation for missing data. All analyses were done using SAS version 9.4. (SAS).

## Results

Invitations to participate were e-mailed to 9779 eligible members of ArthritisPower, and up to three email reminders were sent to non-responders. Emails were opened by 28% (2735/9779) and the registration link was clicked by 25% of those who saw it (683/2735). A total of 538 members agreed to participate and 293 members completed the baseline registration form. Of those, a total of 253 completed baseline PRO assessments. Of the 253 participants, mean age was 56 (SD 9.2) years, 89.3% female, and 91.3% white. The most commonly reported RMD, not mutually exclusive, was OA (64.8%), followed by RA (48.6%), FMS (40.3%), PsA (26.1%), OP (21.0%), AS (15.8%), and SLE (5.9%). Of these, 140 participants (55.3%) completed baseline and all m1–3 PRO assessments for the study within the specified window each month. Attrition was greatest from baseline to m1 (22.9%), when 34 participants completed PRO assessments outside of the required m1 window and 24 were lost to follow-up. Further drop off occurred between m1 to m2 (7.9%) and m2 to m3 (13.8%), when 9 and 18 participants completed PRO assessments outside of the m2 and m3 windows and an additional 11 and 17 participants were lost to follow-up, respectively. No significant differences were found on observed variables when comparing the 140 participants who completed the study versus the 113 who did not (Table 1).

**Table 1:** Baseline demographic and clinical participant characteristics (*N* = 253)

Variable	*N =* 253 (baseline)	*n =* 140 (completers)	*N* = 113 (attriters)
**Age, mean (SD)**	55.7 (9.2)	55.1 (9.3)	56.5 (9.0)
**Female,** ***n*** **(%)**	226 (89.3)	124 (88.6)	102 (90.3)
**Race** ***n*** **(%)**			
White	231 (91.3)	126 (90.0)	105 (92.9)
Black/African American	12 (4.7)	6 (4.3)	6 (5.3)
Other	10 (4.0)	8 (5.7)	2 (1.8)
**Hispanic** ***n*** **(%)**	8 (3.2)	3 (2.1)	5 (4.4)
**Condition,** ***n*** **(%)**			
Osteoarthritis (OA)	164 (64.8)	87 (62.1)	77 (68.1)
Rheumatoid arthritis (RA)	123 (48.6)	67 (47.9)	56 (49.6)
Fibromyalgia syndrome (FMS)	102 (40.3)	56 (40.0)	46 (40.7)
Psoriatic arthritis (PsA)	66 (26.1)	34 (24.3)	32 (28.3)
Osteoporosis (OP)	53 (21.0)	34 (24.3)	19 (16.8)
Ankylosing spondylitis (AS)	40 (15.8)	18 (12.9)	22 (19.5)
Systemic lupus erythematosus (SLE)	15 (5.9)	6 (4.3)	9 (8.0)
**BMI, mean (SD)**	32.2 (8.7)	31.9 (9.1)	32.6 (8.3)
**Number of years living with condition, mean (SD)**	11.6 (10.6)	11.4 (10.8)	11.9 (10.3)
**Married,** ***n*** **(%)**	142 (56.1)	74 (52.9)	68 (60.2)
**Education,** ***n*** **(%)**			
Post-secondary school	230 (90.9)	129 (92.1)	101 (89.4)
**Employment status,** ***n*** **(%)**			
Currently employed (full-, part-time, self-employed)	93 (36.8)	52 (37.1)	41 (36.3)
**Comorbid conditions,** ***n*** **(%)**			
Depression	128 (50.6)	74 (52.9)	54 (47.8)
Hypertension	116 (45.8)	64 (45.7)	52 (46.0)
Hypercholesterolemia	89 (35.2)	49 (35.0)	40 (35.4)
Psoriasis	62 (24.5)	31 (22.1)	31 (27.4)
Diabetes	30 (11.9)	18 (12.9)	12 (10.6)

*T* tests were performed for continuous variables and chi square tests or Fisher’s exact tests for categorical variables to compare the difference between groups of participants who completed and attrited. No statistical significance (*p* < 0.05) was observed

Pain was the most frequently selected symptom to track (83.0%) among baseline participants (*N* = 253), though it was split across two measures available for selection, PROMIS Pain Interference and PROMIS Pain Intensity. PROMIS Fatigue (77.9%) was the single measure selected most often across all participants. PROMIS Physical Function (67.2%) was also a commonly selected single measure. Moreover, at least one of the PROMIS mental health domain instruments (82.2%) was also commonly selected. Among participants with RA (*n* = 123, 48.6% of baseline cohort), more than two thirds (69.9%) chose to track the OMERACT RA Flare instrument at baseline. Most PROs were just as likely to be selected at baseline by those who did and did not complete the study, but attriters were significantly more likely than completers to choose to track anxiety (54.9% vs. 33.6%; *p* < 0.01) (Appendix 3).

To assess whether and how participants changed their PRO selections over time, we examined the 140 participants who completed baseline PRO selection and m1–3 of assessments during the specified time window over the study period (Table 2). Minimal PRO selection changes were observed across the four timepoints. A notable exception was the OMERACT RA Flare instrument as there was a significant decline over time in selection of this measure. Specifically, among the fifty-two of 67 (77.6%) RA participants who selected RA Flare at baseline and completed m1–3, forty-five (67.2%) chose to track it at m1 and thirty-eight (56.7%) at m2, but only ten (14.9%) at m3.

**Table 2:** PRO selections made by study participants, baseline, and m1–3 (completers, *n* = 140)

Symptom	Instrument	Baseline, completers (***N*** = 140)	m1, completers (***N*** = 140)	m2, completers (***N*** = 140)	m3, completers (***N*** = 140)
Pain					
	Completion of ANY Pain Instrument	121 (86.4)	121 (86.4)	120 (85.7)	121 (86.4)
	PROMIS Pain Interference	70 (50.0)	75 (53.6)	76 (54.3)	77 (55.0)
	PROMIS Pain Behavior	53 (37.9)	55 (39.3)	55 (39.3)	56 (40.0)
	PROMIS Pain Intensity	68 (48.6)	73 (52.1)	73 (52.1)	76 (54.3)
Physical function	PROMIS Physical Function	93 (66.4)	94 (67.1)	95 (67.9)	96 (68.6)
Mental health					
	Completion of ANY Mental Health Instrument	115 (82.1)	116 (82.9)	116 (82.9)	116 (82.9)
	PROMIS Depression	76 (54.3)	76 (54.3)	76 (54.3)	76 (54.3)
	PROMIS Anxiety	47 (33.6)	48 (34.3)	49 (35.0)	50 (35.7)
	PROMIS Applied Cognition Abilities	58 (41.4)	60 (42.9)	60 (42.9)	62 (44.3)
	PROMIS Anger	19 (13.6)	19 (13.6)	19 (13.6)	21 (15.0)
Fatigue	PROMIS Fatigue	111 (79.3)	111 (79.3)	113 (80.7)	114 (81.4)
Social health					
	Any Completion of ANY Social Health Instrument	92 (65.7)	94 (67.1)	100 (71.4)	101 (72.1)
	PROMIS Social Isolation	44 (31.4)	45 (32.1)	48 (34.3)	51 (36.4)
	PROMIS Social Sat DSA	19 (13.6)	20 (14.3)	22 (15.7)	22 (15.7)
	PROMIS Satisfaction Roles Activities	12 (8.6)	13 (9.3)	14 (10.0)	14 (10.0)
	PROMIS Ability to Participate Social	45 (32.1)	46 (32.9)	49 (35.0)	49 (35.0)
	PROMIS Emotional Support	17 (12.1)	17 (12.1)	18 (12.9)	19 (13.6)
Sexual function	PROMIS Sexual Function and Satisfaction	11 (7.9)	11 (7.9)	12 (8.6)	12 (8.6)
Sleep	PROMIS Sleep Disturbance	79 (56.4)	80 (57.1)	83 (59.3)	86 (61.4)
Morning joint stiffness	Duration Morning Joint Stiffness	75 (53.6)	77 (55.0)	76 (54.3)	76 (54.3)
RA flare^+^	OMERACT RA Flare Instrument	52 (77.6)	45 (67.2)	38 (56.7)	10 (14.9)

*m1* month 1, *m2* month 2, *m3* month 3, *Social Sat DSA* Satisfaction with Participation in Discretionary Social Activities

Participants were able to select a minimum of 3 and maximum of 10 assessments

^+^Only RA participants were able to select this assessment (*n* = 67 of 140 completers)

**Fig. 1:**
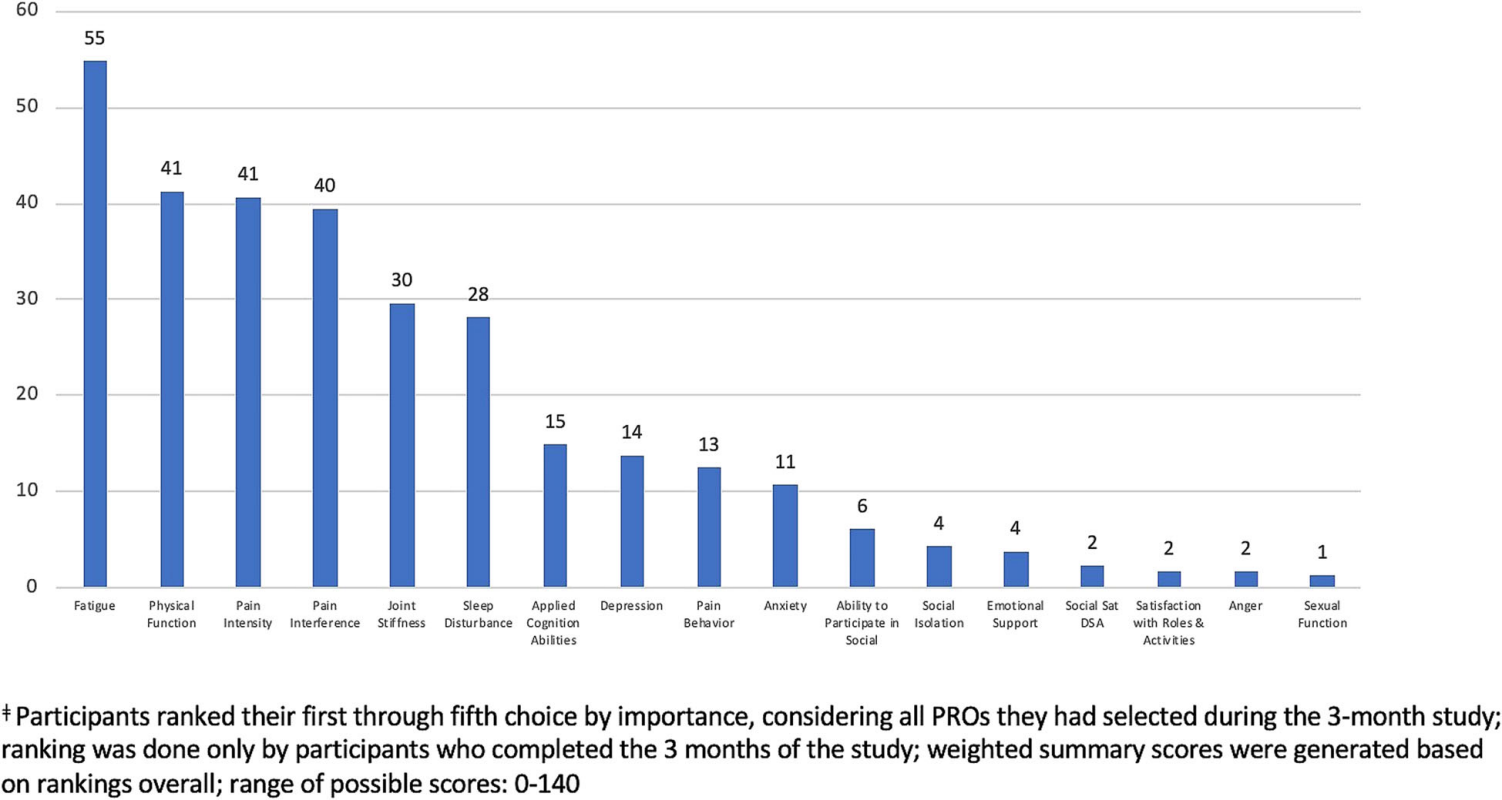
Overall participant ranking of PRO selections at study conclusion (m3), weighted summary score (*n* = 140)

At study conclusion (m3), participants who completed baseline plus m1–3 ranked the PROs they had chosen to track at any point during the study. PROMIS Fatigue had the highest weighted summary score overall (54.8), followed by PROMIS Physical Function (41.3), PROMIS Pain Intensity (40.7), PROMIS Pain Interference (39.5), Duration of Morning Joint Stiffness (29.6), and PROMIS Sleep Disturbance (28.1). PROMIS Satisfaction with Roles and Activities (1.7), PROMIS Anger (1.7), and PROMIS Sexual Function (1.3) ranked lowest overall (Fig. 1). In addition, after calculating the mean (SD) of participants’ rankings for each PRO overall, the same rank-order prioritization of symptoms was observed for the top six: PROMIS Fatigue, 39.2 (35.6); PROMIS Physical Function, 29.5 (33.8); PROMIS Pain Intensity, 29.1 (39.4); PROMIS Pain Interference, 28.2 (36.4); Duration of Morning Joint Stiffness, 21.1 (31.6); and PROMIS Sleep Disturbance, 20.1 (26.7) (Table 3).

**Table 3:** Comparison of PRO prioritization at study conclusion (m3) by condition, weighted rank mean score (standard deviation)

	All (***N*** = 140)	RA (***n*** = 56)	PsA (***n*** = 28)	OA (***n*** = 19)	AS (***n*** = 18)	FMS (***n*** = 15)	SLE (***n*** = 4)
PROMIS Fatigue^*^	39.2 (35.6)	50.0 (37.3)	33.9 (30.2)	21.1 (32.6)	33.8 (31.6)	36.7 (37.2)	43.8 (42.7)
**PROMIS Physical Function**	29.5 (33.8)	26.6 (33.2)	38.1 (41.0)	28.9 (27.6)	25.6 (33.3)	25.2 (27.9)	45.8 (41.7)
**PROMIS Pain Intensity**	29.1 (39.4)	23.3 (37.2)	39.4 (42.0)	34.4 (39.3)	31.4 (44.5)	29.7 (39.3)	0 (0)
**PROMIS Pain Interference**	28.2 (36.4)	25.1 (33.3)	19.8 (33.0)	34.2 (43.5)	39.5 (38.4)	33.0 (39.8)	33.3 (47.1)
**Duration of Morning Joint Stiffness**	21.1 (31.6)	19.2 (30.1)	18.8 (28.7)	28.6 (35.4)	19.0 (32.2)	24.7 (35.0)	25.0 (50.0)
**PROMIS Sleep Disturbance**	20.1 (26.7)	24.3 (30.0)	21.0 (24.4)	16.4 (27.0)	16.6 (27.2)	11.9 (18.9)	19.6 (14.2)
**PROMIS Applied Cognition Abilities**	10.7 (22.4)	12.6 (23.7)	9.6 (20.7)	3.5 (10.5)	15.8 (26.9)	11.3 (28.0)	0 (0)
**PROMIS Depression**	9.9 (20.5)	8.0 (18.0)	7.9 (13.9)	20.3 (31.4)	6.9 (14.4)	13.6 (28.4)	0 (0)

Participants ranked their first through fifth choice by importance, considering all PROs they had selected during the 3-month study; ranking was done only by participants who completed the study; weighted rank mean scores for participants’ ranking of each PRO were generated by using weighted individual participant rankings multiplied by 100, then taking the mean of participants’ rankings for each PRO; PROs unranked by participants carried a zero value for inclusion in mean ranking score calculation; range of possible scores 0–100

^*^Statistical significance (*p <* 0.05) from ANOVA to compare mean PRO ranking scores overall across conditions

We also wanted to evaluate whether PRO prioritization at m3 differed by rheumatic disease. After applying the hierarchy of conditions for mutually exclusive disease subgroups, RA was the most common condition (*n* = 56), followed by PsA (*n* = 28), OA (*n* = 19), AS (*n* = 18), FMS (*n* = 15), and SLE (*n* = 4); no OP participants remained after populating other disease subgroups in the hierarchy (Table 3). The mean of participants’ PRO rankings were similar across conditions, with the exception of PROMIS Fatigue, which had a higher mean ranked score among RA participants than OA participants (50.0 vs. 21.1), a difference that was significant (*p <* 0.05) in the Turkey pairwise comparison.

Among 140 study completers, sixteen opted to suggest other symptoms they would have wanted to track that were not included in this study. These included appetite, dehydration, pain in specific sites (e.g., hand, foot), stress level, and “leg cramps that wake me up at night.” Other suggested items were either already included in the study (i.e., pain, sleep) or included under different measure names than those proposed by participants. For example, whereas participants suggested “concentration/alertness,” and activity level or “exercise abilities,” the study workflow had specified “Applied Cognition Abilities” and “Physical Function.” Finally, although meteorology is not a symptom, three participants suggested “weather” or “weather effects” as something they would have liked to track.

The average number of PROs selected by participants to track each month was stable over the course of the study. Study protocol parameters required that participants select at least 3, but no more than 10 measures, to track at any time. A mean number of 7.0 (SD 2.5) PRO measures were selected at baseline, 6.8 (SD 2.3) at m1, 6.9 (SD 2.4) at m2, and 6.9 (SD 2.5) at m3. The biggest shift was observed among those selecting to track the maximum of 10 at once early in the study; between baseline and m1, there was a 6.7% decrease in frequency of participants choosing to track 10 instruments. ArthritisPower participants took an average (median) of about 30 s to complete each instrument, with time-to-completion ranging from approximately 15 s (Duration of Morning Joint Stiffness) to 70 s (OMERACT RA Flare). Therefore, participants spent a median time of 210 s (7 measures × 30 s), or 3.5 min, per month completing assessments. See Appendix for full details about frequency of participant selection of 3, 4, 5, 6, 7, 8, 9, or 10 assessments at each time period and median and interquartile range for time-to-complete PRO assessments included in the study.

## Discussion

Participants overall preferred selecting measures pertaining to pain, fatigue, and physical function over other measures offered in this study of RMD patients. PRO selections did not vary greatly over time, with the exception of RA flare. Participants’ interest in tracking RA flare declined over the 3-month period from baseline to study conclusion. Though fatigue was considered more important for participants with RA than with OA, across all rheumatic diseases, participants consistently prioritized PROMIS Pain Intensity, Pain Interference, Fatigue, and Physical Function as important symptoms to track.

These findings reinforce and clarify the conclusions of prior studies investigating PROs of interest to RMD patients. The OMERACT work group for RA Flare core domains found that fatigue, pain, and stiffness were key symptoms for RA patients’ experience of disease [28]. Notably, fatigue is a critical symptom concern for patients that may not receive adequate attention in disease management. An earlier Delphi consensus process surveying a patient group and a sample of rheumatology health care providers found agreement among both groups that pain, physical function, and stiffness were important RA symptoms; however, while fatigue was considered very important to patients, it was largely overlooked by providers [29], perhaps because it is less actionable. Although RA clinical trials have examined a variety of symptoms using PROs, most commonly for physical function, pain, and morning joint stiffness [30], domains identified here as important to patients, such as fatigue and sleep disturbance, had been infrequently used in RA trials until recently [31–33].

The sharp decline we observed in participants’ selection of the OMERACT RA Flare instrument, a composite measure with individual items for pain, stiffness, fatigue, and physical function, perhaps indicates a preference among patients to observe changes over time in specific symptoms rather than track their condition with a set of pre-specified symptoms or patients may have an easier time self-assessing their pain or fatigue versus identifying a flare. Patients might also have attempted to eliminate redundancy or minimize time spent answering questions; completing the RA flare measure took twice the time needed to finish a PROMIS symptom measure.

Other factors may have affected patients’ choices. The preference for PRO measures with common names (e.g., Fatigue, Morning Joint Stiffness) may reflect patients’ comfort with familiar terms and concepts when tracking disease. Moreover, patients were guided through a workflow with pre-defined categories to select and confirm what they wanted to track for this study. A recommended improvement for future research in this area would be to let patients pick concepts out of a word cloud and then help them map to specific PRO instruments by name. The appendix figures show what screens were presented to patients; the list of measures and their category headings (i.e., Pain, Physical Health, Mental Health, Social Health, RA) were pre-determined by the study team. Although descriptions were available for each set of PRO options by clicking on “What do these measures mean?,” a future iteration could assign patient-friendly names with recognizable symptoms to measures when an official instrument name is not self-evident to a lay person (e.g., “Brain Fog” in lieu of “PROMIS Applied Cognition Abilities”). In short, future methodological enhancements would revise how symptoms are described, let patients choose from a broader set of symptom descriptions, and then pick instruments for them based on the type of symptoms patients want to track.

These findings also imply that patients consider their condition in terms of primary symptoms and their impacts. Perhaps patients highlight pain, fatigue, and physical function as important because they are immediately attributable to their RMD. Nevertheless, a chronic rheumatic condition may have cascading impacts on some patients’ mental [34, 35] and social health [36]. Thus, some PROs that participants frequently selected to track or prioritize, such as PROMIS Applied Cognition Abilities or Depression, may be byproducts of physical symptoms rather than primary symptoms of RMD. To elucidate this point and to understand participants’ rationale for de-selecting the OMERACT RA Flare instrument over time, future qualitative research in this area should explore reasons that patients choose to document some symptoms and not others.

The entirely virtual nature of this longitudinal study sheds light on future RMD patient research and remote patient monitoring as an essential component of virtual healthcare. Participants received reminders and completed their initial PRO selections and ongoing assessments via smartphone app or web-based equivalent. This represents an innovation in the way that clinical trials and real-world studies can be conducted, demonstrating that a study design with limited to no involvement from clinical sites, and dependent upon patients’ use of now commonplace technology (i.e., smartphones), is feasible. Although this study took place before COVID-19, in an era of expanding technology use and “social distancing” to mitigate the risk of infection, this study demonstrates the capacity and willingness of RMD patients to use an application like ArthritisPower and the potential for its use beyond research, namely for digital health, telemedicine, and remote patient monitoring.

Future app-based longitudinal studies should take steps to optimize participation. In this study, more than two thirds of eligible members never saw the invitation because they never opened the email and, even among those who opened it, there were many who chose not to click on the link in the email to view the full study description. A greater proportion of eligible members might have participated if more had been done to foster enthusiasm for the study, for example with more outreach before the study or with invitation email language that made a stronger case for the importance of participation in the study to help inform and improve care. Nevertheless, given the distribution and mean number of PRO measures participants elected to track each month, it appears that patients living with RMD are interested in tracking multiple symptoms at once and amenable to spending 3 to 5 min completing assessments on a regular basis (e.g., monthly). However, some of the attrition observed between baseline and m1 may have been due to an over-ambitious selection of the maximum number of PROs at baseline by some participants. Longitudinal studies such as this may reasonably expect an attrition rate of above one third of initial study enrollment. In this study, reasons for the high attrition rate included potential confusion about precisely when each month that selected PROs ought to be completed. Moreover, there were no guard rails in the app to prevent participants from completing their assessments early or late each month. This may have caused some participants to believe that they had completed their study tasks for the month and ignore programmed notifications and prompts for this sub-study. Since ArthritisPower participants are typically able to log in and complete PRO assessments on a weekly, or even daily, basis, it will be necessary in a future app workflow of this type to disable the ability to complete assessments outside of the specified monthly window.

The findings of this study should be considered in light of several limitations. Participants in the study were already members of ArthritisPower and therefore users of the ArthritisPower smartphone app or its web-based equivalent, and this may have contributed selection bias. While the participants in this study successfully used the technology, some caution is needed regarding interpreting these findings too broadly. ArthritisPower members self-reported their condition(s) and self-selected their participation in the sub-study to choose they PROs wanted and commit to tracking them over several months. Moreover, as a technology-based registry, the demographics of the sample may not generalize to non-Caucasians and patients with limited access to technology. We also recognize that despite some interesting contrasts between diseases, we had low participant numbers for PsA, AS, SLE, and OP, which limited inference about these subgroups.

## Conclusion

These findings provide insights into the symptoms that rheumatology patients may find most important and will be useful to inform the design of future patient-centric clinical trials, real-world evidence generation, and remote patient monitoring as a component of virtual healthcare.

## Supplementary Information

**Additional file 1.**

## Data Availability

The datasets used and/or analyzed during the current study are available from the corresponding author on reasonable request.

## Abbreviations

GHLF: Global Healthy Living Foundation
PCORI: Patient-Centered Outcomes Research Institute
PRO(s): Patient-reported outcome(s)
pts: Participants
PROMIS: Patient-Reported Outcomes Measurement Information System
RA: Rheumatoid arthritis
OMERACT: Outcome Measures in Rheumatology Clinical Trials
ACR: American College of Rheumatology
EULAR: The European League Against Rheumatism
ICHOM: International Consortium of Health Outcome Measurement
RMD: Rheumatic and musculoskeletal diseases
AS: Ankylosing spondylitis
FMS: Fibromyalgia syndrome
OA: Osteoarthritis
OP: Osteoporosis
PsA: Psoriatic arthritis
SLE: Systemic lupus erythematosus
UAB: University of Alabama at Birmingham
CAT: Computerized adaptive testing
SD: Standard deviation
ANOVA: Analysis of variance
HSD: Honestly significant difference
SAS: SAS Institute
