# Supplementary material for: Which patient-reported outcomes do rheumatology patients find important to track digitally? A real-world longitudinal study in ArthritisPower
Source: Arthritis Res Ther. 2021 Feb 10;23:53. doi: 10.1186/s13075-021-02430-0 (PMC7873115; doi:10.1186/s13075-021-02430-0)
Supplement: Supplementary file 1 — Additional file 1. [file 13075_2021_2430_MOESM1_ESM.docx]

**SUPPLEMENTARY MATERIAL / APPENDIX**

**Contents**

1. (Table) Symptoms and Corresponding PRO Measures Offered for Selection by Study Participants
2. (Figure) Participant Instructions in ArthritisPower App
3. (Table) Comparison of Baseline PRO Selection, Attriters vs. Completers (N=253)
4. (Figure) Number of Participants Ranking Each PRO Among Top 5 Most Important to Track at Study Conclusion (m3) (N=140)
5. (Table) Frequency of Number of Instruments Selected (Count) by Month
6. (Table) Median Time-to-Complete PRO Assessments
7. (Table) PRO Selection at m3 by Condition (not mutually exclusive) (N=140)
8. (Table) Comparison of PRO Prioritization at Study Conclusion (m3) by Condition, Weighted Rank Mean Score (Standard Deviation) (FULL TABLE of Table 3 in main manuscript)

Appendix 1. (Table) Symptoms and Corresponding PRO Measures Offered for Selection by Study Participants

| **Symptom** | **PRO Measure(s)** |
| --- | --- |
| Pain | PROMIS Bank v1.1 – Pain Interference  PROMIS Bank v1.0 – Pain Behavior  PROMIS Bank v1.0 – Pain Intensity |
| Physical Function | PROMIS Bank v1.2 - Physical Function |
| Mental Health | PROMIS Bank v1.0 – Depression  PROMIS Bank v1.0 – Anxiety  PROMIS Bank v1.0 – Applied Cognition Abilities  PROMIS Bank v1.1 – Anger  PROMIS Bank v2.0 – Emotional Support |
| Fatigue | PROMIS Bank v1.0 – Fatigue |
| Social Health | PROMIS Bank v2.0 – Social Isolation  PROMIS Bank v1.0 – Satisfaction with Discretionary Social Activities (DSA)  PROMIS Bank v2.0 – Satisfaction with Roles and Activities  PROMIS Bank v2.0 – Ability to Participate Social |
| Impact on Work | PROMIS Bank v2.0 – Satisfaction Roles Activities |
| Sexual Function | Generic question: “In the past 7 days, to what extent has your condition affected your satisfaction with your sex life?”  PROMIS Bank v1.0 – Sexual Function and Satisfaction: Factors Interfering with Sexual Satisfaction, specifically the following two items:   - SFFAC101: “How much has fatigue or lack of energy affected your satisfaction with your sex life?” - SFFAC102: “How much has pain affected your satisfaction with your sex life?” |
| Sleep | PROMIS Bank v1.0 – Sleep Disturbance |
| Morning Joint Stiffness | Duration of Morning Joint Stiffness* |
| RA Flare | OMERACT RA Flare instrument |

Computerized Adaptive Testing (CAT) versions were used for all PROMIS measures unless otherwise specified.

*Instrument developed by Eli Lilly & Company

**Appendix 2. (Figure) Participant Instructions in ArthritisPower App
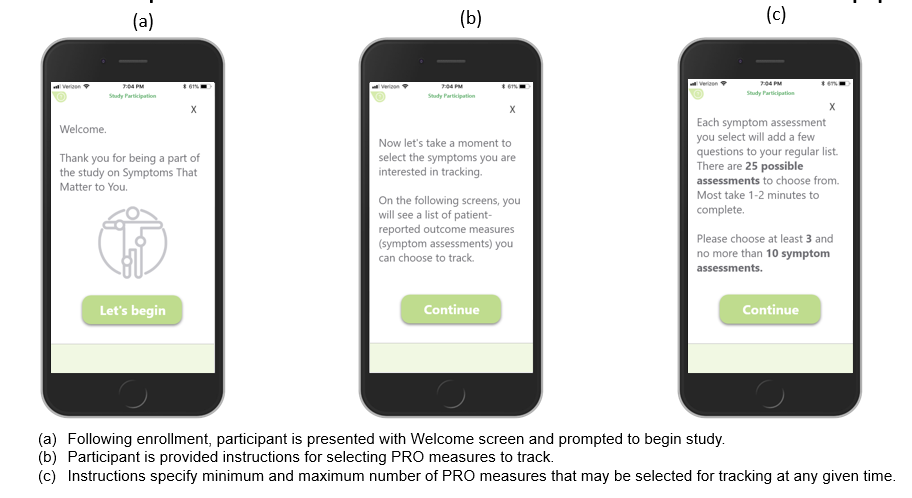
**

**
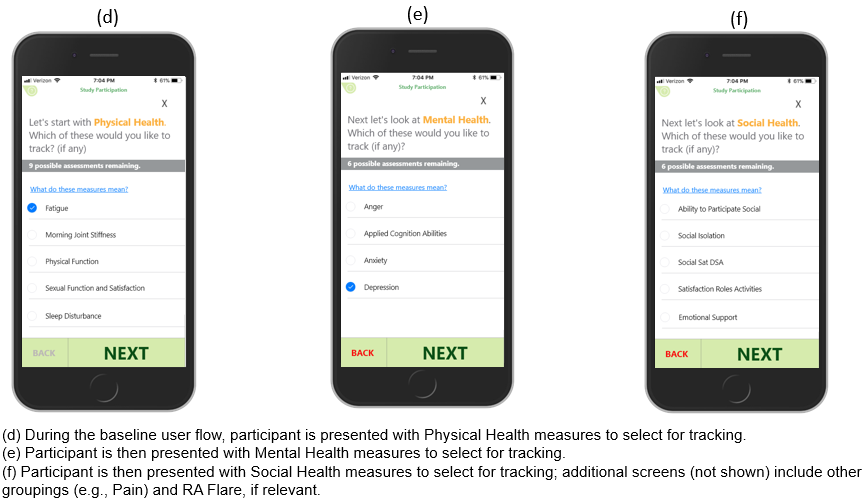

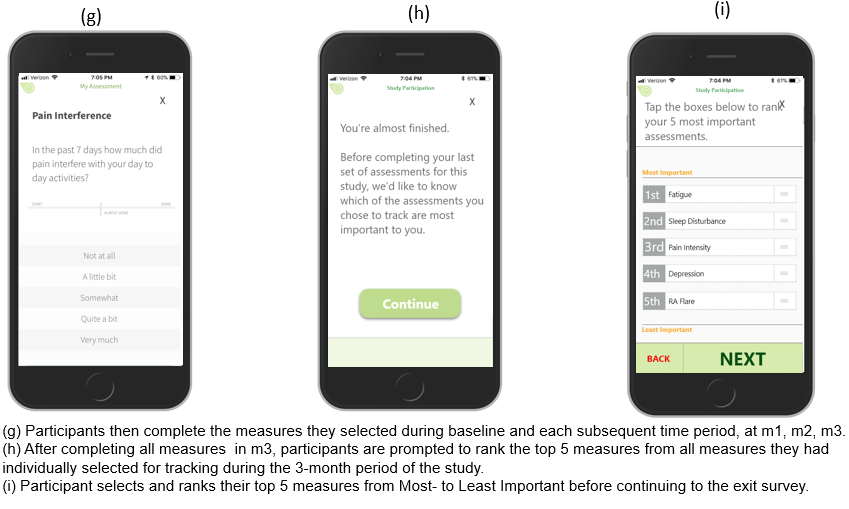
**

**Appendix 3. (Table) Comparison of Baseline PRO Selection**^ǂ^**, Attriters vs. Completers (N=253)**

| **Symptom** | **Instrument** | Baseline (N=253) | Baseline (Study Attriters; N=113) | Baseline (Study Completers; N=140) |
| --- | --- | --- | --- | --- |
| Pain |  |  |  |  |
|  | Completion of ANY Pain Instrument | 210 (83.0) | 89 (78.8) | 121 (86.4) |
|  | PROMIS Pain Interference | 117 (46.3) | 47 (41.6) | 70 (50.0) |
|  | PROMIS Pain Behavior | 105 (41.5) | 52 (46.0) | 53 (37.9) |
|  | PROMIS Pain Intensity | 122 (48.2) | 54 (47.8) | 68 (48.6) |
| Physical Function | PROMIS Physical Function | 170 (67.2) | 77 (68.1) | 93 (66.4) |
| Mental Health |  |  |  |  |
|  | Completion of ANY Mental Health Instrument | 208 (82.2) | 93 (82.3) | 115 (82.1) |
|  | PROMIS Depression | 132 (52.2) | 56 (49.6) | 76 (54.3) |
|  | PROMIS Anxiety* | 109 (43.1) | 62 (54.9) | 47 (33.6) |
|  | PROMIS Applied Cognition Abilities | 100 (39.5) | 42 (37.2) | 58 (41.4) |
|  | PROMIS Anger | 38 (15.0) | 19 (16.8) | 19 (13.6) |
| Fatigue | PROMIS Fatigue | 197 (77.9) | 86 (76.1) | 111 (79.3) |
| Social Health |  |  |  |  |
|  | Any Completion of ANY Social Health Instrument | 168 (66.4) | 76 (67.3) | 92 (65.7) |
|  | PROMIS Social Isolation | 80 (31.6) | 36 (31.9) | 44 (31.4) |
|  | PROMIS Social Sat DSA | 36 (14.2) | 17 (15.0) | 19 (13.6) |
|  | PROMIS Satisfaction Roles Activities | 22 (8.7) | 10 (8.9) | 12 (8.6) |
|  | PROMIS Ability to Participate Social | 75 (29.6) | 30 (26.6) | 45 (32.1) |
|  | PROMIS Emotional Support | 36 (14.2) | 19 (16.8) | 17 (12.1) |
| Sexual Function | PROMIS Sexual Function and Satisfaction | 25 (9.9) | 14 (12.4) | 11 (7.9) |
| Sleep | PROMIS Sleep Disturbance | 152 (60.1) | 73 (64.6) | 79 (56.4) |
| Morning Joint Stiffness | Duration of Morning Joint Stiffness | 141 (55.7) | 66 (58.4) | 75 (53.6) |
| RA Flare^+^ | OMERACT RA Flare Instrument | 86 (69.9) | 34 (30.1) | 52 (77.6) |

Social Sat DSA: Satisfaction with Participation in Discretionary Social Activities

*Statistical significance (p < 0.05) for chi square tests between groups of participants who completed and attrited.

^ǂ^Participants were able to select a minimum of 3 and maximum of 10 assessments

^+^Only RA participants were able to select this assessment (N=123, baseline)

**Appendix 4. (Figure) Number of Participants Ranking PRO Among Their Top 5 Most Important to Track at Study Conclusion (m3) (N=140)**


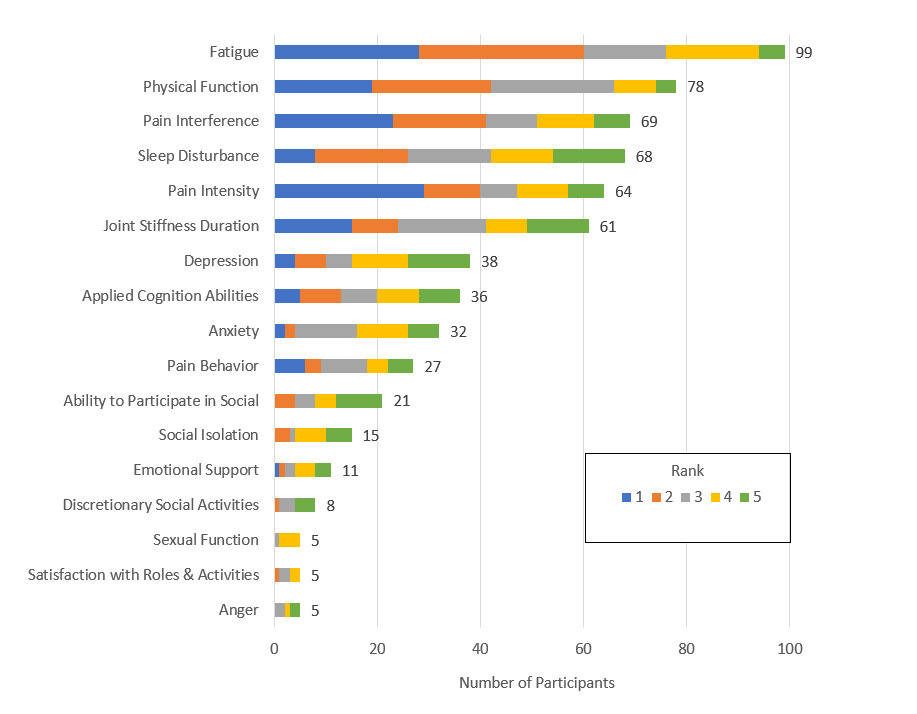


PRO: patient-reported outcome measure; m3: Month 3

**Appendix 5. (Table) Frequency of Number of Instruments Selected (Count) by Month**

| **Count** | **Baseline (N=253)** | **Month 1 (N=195)** | **Month 2 (N=175)** | **Month 3 (N=140)** |
| --- | --- | --- | --- | --- |
| 3 | 25 (9.9) | 15 (7.7) | 11 (6.3) | 15 (10.7) |
| 4 | 34 (13.4) | 28 (14.4) | 28 (16.0) | 18 (12.9) |
| 5 | 25 (9.9) | 23 (11.8) | 18 (10.3) | 12 (8.6) |
| 6 | 18 (7.1) | 22 (11.3) | 21 (12.0) | 17 (12.1) |
| 7 | 33 (13.0) | 26 (13.3) | 21 (12.0) | 14 (10.0) |
| 8 | 26 (10.3) | 21 (10.8) | 17 (9.7) | 17 (12.1) |
| 9 | 36 (14.2) | 30 (15.4) | 29 (16.6) | 19 (13.6) |
| 10 | 56 (22.1) | 30 (15.4) | 30 (17.1) | 28 (20.0) |

**Appendix 6. (Table) Median Time-to-Complete PRO Assessments**

| **PRO Assessments** | **Median Time-to-Complete,**  **seconds (IQR)** |
| --- | --- |
| Pain |  |
| Pain Interference | 30 (24-43) |
| Pain Behavior | 28 (22-38) |
| Pain Intensity | 17 (14-22) |
| Physical Function | 34 (28-43) |
| Mental Health |  |
| Depression | 23 (18-35) |
| Anxiety | 25 (20-36) |
| Applied Cognition Abilities | 28 (22-39) |
| Anger | 33 (26-47) |
| Fatigue | 31 (26-39) |
| Social Health |  |
| Social Isolation | 23 (19-33) |
| Social Satisfaction Discretionary Social Activities | 35 (26-54) |
| Satisfaction Roles Activities | 31 (24-44) |
| Ability to Participate Social | 29 (23-44) |
| Emotional Support | 25 (20-38) |
| Sexual Function | 28 (21-126) |
| Sleep Disturbance | 29 (23-41) |
| Duration of Morning Joint Stiffness | 15 (11-23) |
| OMERACT RA Flare | 70 (58-92) |

IQR: Interquartile range

**Appendix 7. (Table) PRO Selection^ƚ^ at m3 by Condition (not mutually exclusive) (N=140)**

| **Symptom** | **Instrument** | Total (N=140) | OA (N=87) | RA (N=67) | FMS (N=56) | OP (N=34) | PsA (N=34) | AS (N=18) | SLE (N=6) |
| --- | --- | --- | --- | --- | --- | --- | --- | --- | --- |
| Pain |  |  |  |  |  |  |  |  |  |
|  | Completion of ANY Pain Instrument | 121 (86.4) | 78 (89.7) | 56 (83.6) | 51 (91.1) | 29 (85.3) | 32 (94.1) | 16 (88.9) | 4 (66.7) |
|  | PROMIS Pain Interference | 77 (55.0) | 51 (58.6) | 35 (52.2) | 39 (69.6) | 22 (64.7) | 20 (58.8) | 14 (77.8) | 2 (33.3) |
|  | PROMIS Pain Behavior | 56 (40.0) | 37 (42.5) | 25 (37.3) | 23 (41.1) | 14 (41.2) | 13 (38.2) | 8 (44.4) | 2 (33.3) |
|  | PROMIS Pain Intensity | 76 (54.3) | 50 (57.5) | 31 (46.3) | 33 (58.9) | 20 (58.8) | 23 (67.6) | 10 (55.6) | 1 (16.7) |
| Physical Health | PROMIS Physical Function | 96 (68.6) | 61 (70.1) | 44 (65.7) | 40 (71.4) | 22 (64.7) | 26 (76.5) | 12 (66.7) | 3 (50.0) |
| Mental Health |  |  |  |  |  |  |  |  |  |
|  | Completion of ANY Mental Health Instrument | 116 (82.9) | 73 (83.9) | 53 (79.1) | 50 (89.3) | 28 (82.4) | 30 (88.2) | 17 (94.4) | 5 (83.3) |
|  | PROMIS Depression | 76 (54.3) | 51 (58.6) | 29 (43.3) | 37 (66.1) | 18 (52.9) | 18 (52.9) | 15 (83.3) | 2 (33.3) |
|  | PROMIS Anxiety | 50 (35.7) | 27 (31.0) | 21 (31.3) | 22 (39.3) | 11 (32.4) | 14 (41.2) | 7 (38.9) | 3 (50.0) |
|  | PROMIS Applied Cognition Abilities | 62 (44.3) | 38 (43.7) | 30 (44.8) | 31 (55.4) | 16 (47.1) | 16 (47.1) | 11 (61.1) | 2 (33.3) |
|  | PROMIS Anger | 21 (15.0) | 11 (12.6) | 13 (19.4) | 13 (23.2) | 7 (20.6) | 3 (8.8) | 5 (27.8) | 2 (33.3) |
| Fatigue | PROMIS Fatigue | 114 (81.4) | 71 (81.6) | 58 (86.6) | 50 (89.3) | 30 (88.2) | 29 (85.3) | 15 (83.3) | 5 (83.3) |
| Social Health |  |  |  |  |  |  |  |  |  |
|  | Any Completion of ANY Social Health Instrument | 101 (72.1) | 61 (70.1) | 46 (68.7) | 40 (71.4) | 29 (85.3) | 28 (82.4) | 13 (72.2) | 6 (100) |
|  | PROMIS Social Isolation | 51 (36.4) | 33 (37.9) | 24 (35.8) | 29 (51.8) | 14 (41.2) | 14 (41.2) | 11 (61.1) | 3 (50.0) |
|  | PROMIS Social Sat DSA | 22 (15.7) | 14 (16.1) | 6 (9.0) | 6 (10.7) | 8 (23.5) | 7 (20.6) | 3 (16.7) | 1 (16.7) |
|  | PROMIS Satisfaction Roles Activities | 14 (10.0) | 7 (8.1) | 7 (10.5) | 2 (3.6) | 5 (14.7) | 2 (5.9) | 3 (16.7) | 0 (0.0) |
|  | PROMIS Ability to Participate Social | 49 (35.0) | 24 (27.6) | 25 (37.3) | 16 (28.6) | 13 (38.2) | 14 (41.2) | 3 (16.7) | 2 (33.3) |
|  | PROMIS Emotional Support | 19 (13.6) | 14 (16.1) | 11 (16.4) | 5 (8.9) | 4 (11.8) | 3 (8.8) | 1 (5.6) | 1 (16.7) |
| Sexual Function | PROMIS Sexual Function and Satisfaction | 12 (8.0) | 7 (8.1) | 2 (3.0) | 8 (14.3) | 3 (8.8) | 6 (17.7) | 5 (27.8) | 1 (16.7) |
| Sleep | PROMIS Sleep Disturbance | 86 (61.4) | 54 (62.1) | 40 (59.7) | 34 (60.7) | 25 (73.5) | 22 (64.7) | 10 (55.6) | 5 (83.3) |
| Morning Joint Stiffness | Duration Morning Joint Stiffness | 76 (54.3) | 54 (62.1) | 34 (50.7) | 30 (53.6) | 21 (61.8) | 17 (50.0) | 9 (50.0) | 1 (16.7) |
| RA Flare^+^ | OMERACT RA Flare Instrument | 10 (14.9) | - | 10 (14.9) | - | - | - | - | - |

Social Sat DSA: Satisfaction with Participation in Discretionary Social Activities

**^ƚ^**Participants were able to select a maximum of 10 assessments

^+^Only RA participants were able to select this assessment (n=67)

**Appendix 8. (Table) Comparison of PRO Prioritization^ƚ^ at Study Conclusion (m3) by Condition, Weighted Rank Mean Score (Standard Deviation)**

|  | Total (N=140) | RA (n=56) | PsA (n=28) | OA (n=19) | AS (n=18) | FMS (n=15) | SLE (n=4) |
| --- | --- | --- | --- | --- | --- | --- | --- |
| Fatigue* | 39.2 (35.6) | 50.0 (37.3) | 33.9 (30.2) | 21.1 (32.6) | 33.8 (31.6) | 36.7 (37.2) | 43.8 (42.7) |
| Physical Function | 29.5 (33.8) | 26.6 (33.2) | 38.1 (41.0) | 28.9 (27.6) | 25.6 (33.3) | 25.2 (27.9) | 45.8 (41.7) |
| Pain Intensity | 29.1 (39.4) | 23.3 (37.2) | 39.4 (42.0) | 34.4 (39.3) | 31.4 (44.5) | 29.7 (39.3) | 0 (0) |
| Pain Interference | 28.2 (36.4) | 25.1 (33.3) | 19.8 (33.0) | 34.2 (43.5) | 39.5 (38.4) | 33.0 (39.8) | 33.3 (47.1) |
| Joint Stiffness | 21.1 (31.6) | 19.2 (30.1) | 18.8 (28.7) | 28.6 (35.4) | 19.0 (32.2) | 24.7 (35.0) | 25.0 (50.0) |
| Sleep Disturbance | 20.1 (26.7) | 24.3 (30.0) | 21.0 (24.4) | 16.4 (27.0) | 16.6 (27.2) | 11.9 (18.9) | 19.6 (14.2) |
| Applied Cognition Abilities | 10.7 (22.4) | 12.6 (23.7) | 9.6 (20.7) | 3.5 (10.5) | 15.8 (26.9) | 11.3 (28.0) | 0 (0) |
| Depression | 9.9 (20.5) | 8.0 (18.0) | 7.9 (13.9) | 20.3 (31.4) | 6.9 (14.4) | 13.6 (28.4) | 0 (0) |
| Pain Behavior | 8.9 (22.6) | 9.7 (24.6) | 8.6 (22.2) | 9.1 (14.3) | 7.4 (24.4) | 10.6 (26.8) | 0 (0) |
| Anxiety | 7.6 (16.7) | 4.6 (11.8) | 12.6 (26.9) | 8.2 (12.9) | 8.3 (14.0) | 8.6 (15.9) | 6.3 (12.5) |
| Ability to Participate in Social Roles and Activities | 4.4 (11.3) | 3.5 (9.9) | 4.7 (10.6) | 6.1 (13.4) | 3.0 (8.9) | 4.7 (13.6) | 12.5 (25.0) |
| Social Isolation | 3.1 (9.7) | 3.0 (9.5) | 2.7 (7.9) | 2.6 (11.5) | 6.4 (13.7) | 0 (0.0) | 6.3 (12.5) |
| Emotional Support | 2.7 (11.2) | 2.4 (8.0) | 1.6 (5.9) | 5.3 (22.9) | 1.1 (4.7) | 1.7 (6.5) | 12.5 (25.0) |
| Social Sat DSA | 1.6 (7.1) | 0.4 (2.7) | 3.0 (11.2) | 1.1 (4.6) | 1.1 (4.7) | 3.6 (9.7) | 8.3 (16.7) |
| Satisfaction with Participation in Social Roles and Activities | 1.2 (6.4) | 2.4 (9.1) | 0 (0) | 1.8 (7.6) | 0 (0) | 0 (0.0) | 0 (0) |
| Sexual Function* | 1.0 (5.0) | 0 (0) | 0.9 (4.7) | 0 (0.0) | 4.6 (10.8) | 1.7 (6.5) | 0 (0) |
| Anger | 0.9 (5.0) | 1.0 (5.2) | 2.1 (7.7) | 0 (0.0) | 0 (0) | 0 (0.0) | 5.0 (10.0) |

m1: Month 1; m2: Month 2; m3: Month 3; Social Sat DSA: Satisfaction with Participation in Discretionary Social Activities

**^ƚ^**Participants ranked their first through fifth choice by importance, considering all PROs they had selected during the 3-month study; ranking was done only by participants who completed the study; weighted rank mean scores for participants’ ranking of each PRO were generated by using weighted individual participant rankings multiplied by 100, then taking the mean of participants’ rankings for each PRO; PROs unranked by participants carried a zero value for inclusion in mean ranking score calculation; range of possible scores: 0-100.

* Statistical significance (p < 0.05) from ANOVA to compare mean PRO ranking scores overall across conditions.
